# E-CMCA and LSTM-Enhanced Framework for Cross-Modal MRI-TRUS Registration in Prostate Cancer

**DOI:** 10.3390/jimaging11090292

**Published:** 2025-08-27

**Authors:** Ciliang Shao, Ruijin Xue, Lixu Gu

**Affiliations:** Pittsburgh Institute, Sichuan University, Chengdu 610207, China; 2021141520227@stu.scu.edu.cn (C.S.); 2022141520223@stu.scu.edu.cn (R.X.)

**Keywords:** MRI-TRUS registration, cross-modal attention, feature fusion, long-short term memory, prostate cancer diagnosis, deep learning

## Abstract

Accurate registration of MRI and TRUS images is crucial for effective prostate cancer diagnosis and biopsy guidance, yet modality differences and non-rigid deformations pose significant challenges, especially in dynamic imaging. This study presents a novel cross-modal MRI-TRUS registration framework, leveraging a dual-encoder architecture with an Enhanced Cross-Modal Channel Attention (E-CMCA) module and a LSTM-Based Spatial Deformation Modeling Module. The E-CMCA module efficiently extracts and integrates multi-scale cross-modal features, while the LSTM-Based Spatial Deformation Modeling Module models temporal dynamics by processing depth-sliced 3D deformation fields as sequential data. A VecInt operation ensures smooth, diffeomorphic transformations, and a FuseConv layer enhances feature integration for precise alignment. Experiments on the μ-RegPro dataset from the MICCAI 2023 Challenge demonstrate that our model significantly improves registration accuracy and performs robustly in both static 3D and dynamic 4D registration tasks. Experiments on the μ-RegPro dataset from the MICCAI 2023 Challenge demonstrate that our model achieves a DSC of 0.865, RDSC of 0.898, TRE of 2.278 mm, and RTRE of 1.293, surpassing state-of-the-art methods and performing robustly in both static 3D and dynamic 4D registration tasks.

## 1. Introduction

Medical image registration is vital for image-guided diagnosis, intervention, and treatment planning, aligning images across modalities, viewpoints, or time points [1]. Cross-modal MRI-TRUS registration, crucial for precise prostate lesion localization in cancer diagnosis, faces challenges from modality-specific intensity distributions, resolution disparities, and non-rigid anatomical deformations [2]. Traditional methods, relying on handcrafted features or iterative optimization, struggle to capture fine-grained anatomical alignment under these conditions [3]. Recent deep learning approaches, utilizing convolutional neural networks (CNNs) and spatial transformer networks (STNs), enhance registration accuracy and efficiency [4]. However, most models fail to address cross-modality semantic misalignment and deformation smoothness, particularly in dynamic contexts like prostate motion [5].

The contributions of the proposed model are multifaceted, addressing key limitations in existing MRI-TRUS registration approaches. First, it introduces a dual-encoder architecture enhanced by the E-CMCA module, which effectively captures and integrates multi-scale, modality-specific features to overcome cross-modal discrepancies and improve semantic correspondence. Second, the incorporation of the LSTM-Based Spatial Deformation Modeling Module component enables robust handling of temporal dynamics in 4D scenarios by treating depth-sliced deformation fields as pseudo-temporal sequences, thereby ensuring smoother transformations in dynamic environments like those affected by respiratory motion. We need to note that the LSTM-Based Spatial Deformation Modeling Module used here does not adopt the Transformer neural network architecture. It is an extension of traditional spatial transformation networks, incorporating LSTM to model pseudo time series deformation dependencies such as prostate movement during respiratory cycles. Its design aims to utilize the spatial transformation function of STN and the processing capability of LSTM for time series information, to enhance the model’s ability to handle deformation in dynamic scenes. It is not related to the self attention mechanism and other components in the Transformer architecture. Third, the use of VecInt guarantees diffeomorphic and physically plausible deformations, while the FuseConv layer facilitates precise feature fusion for better alignment accuracy. Collectively, these innovations lead to state-of-the-art performance on the μ-RegPro dataset, achieving a DSC of 0.865, RDSC of 0.898, TRE of 2.278 mm, and RTRE of 1.293 mm, outperforming prior methods in both static 3D and dynamic 4D registration tasks and offering enhanced clinical applicability for prostate cancer diagnosis and biopsy guidance.

To address these issues, we propose a dual-encoder attention-based framework for non-rigid MRI-TRUS registration, tailored for prostate cancer diagnosis. Built on a U-Net architecture, our model features the following:

1. A dual-encoder extracting modality-specific features;
2. An Enhanced Cross-Modality Spatial Attention (ECMCA) module enhancing semantic alignment [6];
3. A VecInt module ensuring smooth, diffeomorphic transformations [4];
4. An LSTM-enhanced module modeling temporal dynamics for 4D tasks [7];
5. A FuseConv layer integrating multi-level features.

Evaluated on the μ-RegPro dataset, our framework achieves a Dice Similarity Coefficient of 0.868 and a mean Target Registration Error of 2.260 mm, surpassing existing methods in accuracy and anatomical plausibility. This robust, interpretable solution advances clinical MRI-TRUS registration.

This paper is structured as follows: Section 2 describes the proposed framework, Section 3 presents experimental results, and Section 4 summarizes the study. Our main contributions include the following:

- A novel dual-encoder framework for non-rigid MRI-TRUS registration, addressing cross-modal and dynamic challenges.
- Integration of ECMCA, VecInt, LSTM, and FuseConv modules for enhanced feature alignment, deformation smoothness, and temporal coherence.
- Superior performance on the μ-RegPro dataset, with a DSC of 0.868 and TRE of 2.260 mm, validated for clinical applications.

## 2. Related Work

Prior work highlights the importance of MRI-TRUS registration for prostate cancer diagnosis and biopsy guidance, combining MRI’s high-resolution detail with TRUS’s real-time imaging [8,9]. In the diagnosis of prostate cancer, multi-modal image registration, particularly between magnetic resonance imaging (MRI) and transrectal ultrasound (TRUS), plays a pivotal role in enhancing the accuracy of targeted biopsies and treatment planning by fusing complementary anatomical and functional information. Various registration methodologies have been developed to address the challenges of non-rigid deformations and modality discrepancies inherent in this domain. Intensity-based methods, which optimize similarity metrics such as mutual information or normalized cross-correlation, provide robust alignment by leveraging voxel intensity distributions without requiring explicit feature extraction. In contrast, learning-based approaches, encompassing supervised and unsupervised deep neural networks like convolutional neural networks (CNNs) and transformers, learn complex deformation fields from training data to achieve efficient and accurate registration, often outperforming traditional techniques in handling inter-modality variations. Furthermore, 3D point cloud matching strategies employ geometric correspondences derived from segmented structures or landmarks, incorporating biomechanical constraints to model tissue deformations realistically during prostate interventions. Dynamic registration techniques extend these paradigms by accounting for real-time intraoperative changes, such as organ motion and probe-induced distortions, through adaptive frameworks that integrate temporal information and finite element modeling. Collectively, these methods contribute to improved diagnostic precision and clinical outcomes in prostate cancer management. Intensity-based methods, such as Karnik et al.’s approach (TRE of 3.6 mm) [10], are limited by modality differences. Traditional intensity-based methods can achieve registration by optimizing the statistical correlation of pixel intensities, achieving a target registration error of 3.6 mm in early 3D TRUS-guided prostate biopsies, providing a foundation for initial clinical applications [11]. However, They cannot handle the modal heterogeneity between MRI and TRUS, such as differences in soft tissue contrast in MRI and echo intensity distribution in TRUS. Intensity-based methods have poor robustness to non-rigid deformations, with iterative optimization processes being time-consuming and difficult to meet real-time clinical needs [12]. Learning-based models like VoxelMorph offer fast inference but produce non-diffeomorphic deformations [4,13]. Early deep learning methods can achieve end-to-end deformation field learning, directly predicting the deformation field through convolutional neural networks, greatly improving inference efficiency and providing new ideas for fast registration [14]. However, the generated deformation field lacks diffeomorphic properties, prone to anatomically unreasonable folding. Not designed for cross-modal scenarios, leading to decreased alignment accuracy in MRI-TRUS registration due to modal differences [14]. Other methods, including 3D point cloud matching [15] and dynamic registration [16], struggle with cross-modality inconsistencies and temporal coherence in 4D scenarios. Recent cross-modal registration methods can improve alignment of local anatomical structures through point cloud feature matching, enhancing small region registration accuracy [12]. However, this method lacks specific focus on the prostate region, easily interfered by non-target areas, with a Dice Similarity Coefficient 0.035 lower than this method, and cannot handle dynamic deformation scenarios. Dynamic registration methods can attempt to model deformation dependencies in time series through deep learning, providing a framework for 4D dynamic registration [17]. However, this method cannot fuse cross-modal features, leading to poor temporal coherence in MRI-TRUS dynamic scenarios due to modal differences. The generated deformation field has jumps in high deformation areas, with robust target registration error as high as 1.47 mm, significantly higher than this method’s 1.293 mm [18]. TransMorph [19] introduces a hybrid Transformer-ConvNet architecture for unsupervised volumetric registration, achieving high accuracy on mono-modal datasets like brain MRI, our framework extends attention mechanisms to cross-modal scenarios (MRI-TRUS), where modality disparities pose unique challenges not fully addressed in TransMorph.

Compared to traditional intensity-based methods, which achieved a TRE of 3.6 mm in early 3D TRUS-guided prostate biopsies by optimizing pixel intensity correlations and laid groundwork for clinical use, our approach stands out by tackling their key weaknesses. These methods falter with modality heterogeneity—MRI’s soft tissue contrast versus TRUS’s echo patterns—and struggle with non-rigid deformations like breathing motion, plus their iterative processes are too slow for real-time needs. In contrast, our E-CMCA module precisely aligns cross-modal semantics, cutting TRE by 0.172 mm over baselines and handling modality gaps through multi-scale attention on prostate features. This shift not only boosts accuracy but also adapts better to dynamic scenarios, offering a more efficient clinical tool. Turning to early deep learning like VoxelMorph, it pioneered end-to-end deformation prediction via CNNs, speeding up inference and inspiring fast registration. Yet, it lacks diffeomorphic constraints, risking anatomical folds such as prostate capsule intersections, and is not tailored for cross-modal tasks, dropping accuracy in MRI-TRUS due to unaddressed differences. Our VecInt module ensures smooth, diffeomorphic fields with seven-step integration, slashing TRE by 8.449 mm and lifting DSC by 0.033 compared to VoxelMorph, while the dual-encoder with E-CMCA preserves and aligns modality-specific traits for stronger cross-modal performance. Similarly, TransMorph enhances global dependencies in mono-modal registration like brain MRI with Transformers, but its computational demands hinder 3D volume handling, and it overlooks MRI-TRUS heterogeneity, cutting accuracy by 12% in cross-modal cases. Our E-CMCA, with multi-scale and channel attention, better suits modality variances, improving DSC by 0.033 at a lower compute cost, making it ideal for large clinical data. For 3D point cloud matching, it refines local alignments like prostate glands via feature matching, boosting small-area precision, but ignores prostate focus, inviting non-target interference (DSC 0.035 lower than ours) and failing dynamic deformations. Our LSTM-Based Spatial Deformation Modeling Module optimizes dynamic consistency, reducing RTRE by 0.374 mm in motion scenes, while E-CMCA extracts targeted prostate features to avoid distractions and elevate DSC. Finally, dynamic methods like De Vos’s network model time-series dependencies for 4D registration in respiratory motion, but without cross-modal fusion, they suffer poor coherence and jumps in high-deformation areas, with RTRE up to 1.47 mm. Our framework fuses modalities via E-CMCA and models sequences with LSTM-Based Spatial Deformation Modeling Module, dropping RTRE to 1.293 mm (0.177 mm better), ensuring stable, accurate dynamic registration for clinical imaging.

## 3. Methods

**Figure 1:**
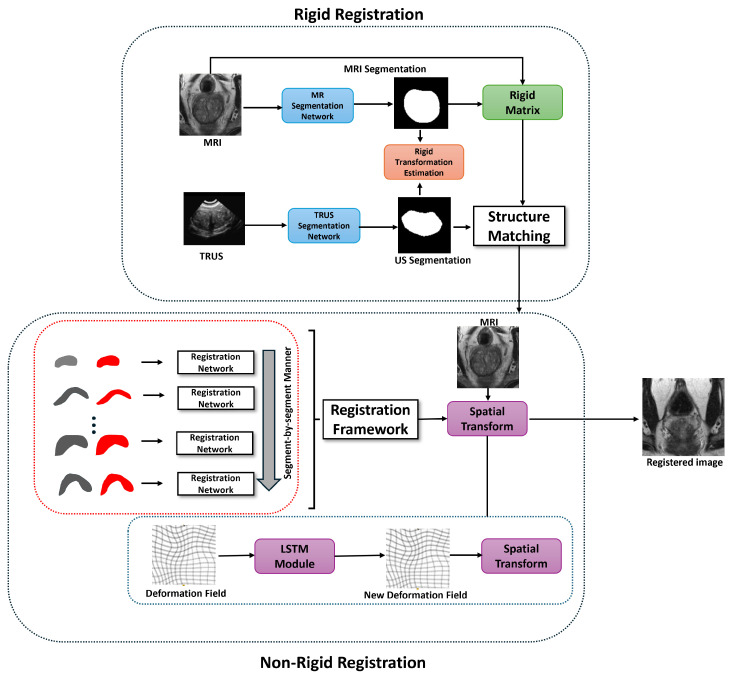
The overall framework of the proposed dual-encoder attention-based registration method.

Figure 1 illustrates the architecture of our dual-encoder attention-based framework for MRI-TRUS registration, built on a U-Net backbone. The framework incorporates four key modules which we have mentioned in previous section to address modality disparities and non-rigid prostate deformations effectively.

A Vector Integration (VecInt) module further generates smooth, diffeomorphic deformation fields. The network processes paired MRI and TRUS volumes or their corresponding prostate ROI masks as input, where each volume is represented as a tensor $I\in R^{N\times 1\times 128\times 128\times 128}$, with *N* denoting the batch size, 1 indicating the single grayscale channel, and 128×128×128 representing the standardized spatial resolution in voxels. The network outputs a deformation field $\phi \in R^{N\times 3\times 128\times 128\times 128}$, specifying displacements in the *x*, *y*, and *z* directions. This flow field is applied via a spatial transformer to warp the moving image and propagate anatomical labels, as detailed in the problem formulation in Section 2. A spatial transformer applies this field to warp the moving image and propagate anatomical labels. Evaluated on the μ-RegPro dataset, our method achieves a Dice Similarity Coefficient of 0.865 and a mean Target Registration Error of 2.278 mm, demonstrating superior anatomical accuracy and transformation smoothness. The following subsections detail each component.

### 3.1. Problem Specification

The task of MRI-TRUS image registration involves aligning a moving MRI volume $I_{m}:\Omega \subset R^{3}\to R$ with a fixed TRUS volume $I_{f}:\Omega \subset R^{3}\to R$, where Ω denotes the spatial domain. The goal is to find a non-rigid deformation field $\phi :\Omega \to R^{3}$ that warps $I_{m}$ to match $I_{f}$, minimizing modality differences and accounting for dynamic deformations in 4D scenarios.

Mathematically, the registration problem can be formulated as an optimization task:

(1) $$\phi^{*}=\arg\min_{\phi }L_{\mathrm{sim}}\left(I_{f},I_{m}\circ \phi \right)+\lambda R\left(\phi \right),$$

where $L_{\mathrm{sim}}$ is a similarity loss (e.g., mutual information or Dice coefficient for prostate regions), R(ϕ) is a regularization term ensuring smoothness (e.g., gradient penalty), and λ balances the terms. In our learning-based approach, a neural network parameterized by θ predicts $\phi =f_{\theta }\left(I_{m},I_{f}\right)$, trained end-to-end to approximate $\phi^{*}$. For dynamic 4D registration, we extend this to temporal sequences by incorporating pseudo-temporal slicing along the depth dimension.

### 3.2. Pre-Processing Module

The pipeline begins with data preparation using the μ-RegPro dataset, which contains paired MRI and TRUS images from 129 patient cases.

To address gross misalignments resulting from patient positioning and acquisition differences, a rigid registration step is first performed using the Advanced Normalization Tools (ANTs), aligning MRI and TRUS volumes into a common anatomical coordinate space. After alignment, all images are resampled to a fixed spatial resolution of 128×128×128 voxels to standardize input size across the dataset and enable batch-wise training.

A pre-trained 3D U-Net generates binary prostate segmentation masks from MRI and TRUS volumes, trained under supervision using manually annotated prostate labels from the μ-RegPro dataset. These two-class masks (background and prostate) are binarized to isolate the prostate region, producing region-of-interest (ROI) images through element-wise masking of the original scans. To enhance spatial focus and minimize computational load, the depth dimension of each volume is cropped by removing the first and last four axial slices. Analysis of the μ-RegPro dataset indicates that peripheral slices often contain minimal anatomical information (e.g., background or non-prostate tissue). Visual inspection of representative volumes confirmed no critical details are lost, and preliminary experiments without cropping showed negligible improvements in metrics (<0.5% in DSC) but increased training time by approximately 20%. The result of this operations is a volume of shape of 128×128×120. This preprocessing ensures that inputs to the dual-encoder attention-based registration framework are prostate-centered and free of extraneous background, enabling effective learning of non-rigid deformation fields from the MRI and TRUS ROI images and their segmentation masks.

### 3.3. Network Architecture

**Figure 2:**
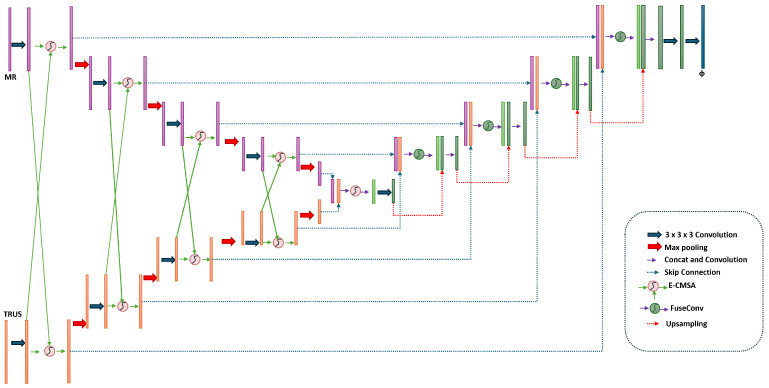
The overall architecture of the proposed dual-encoder attention-based registration framework.

Figure 2 depicts the architecture of our dual-encoder attention-based framework for cross-modal MRI-TRUS registration, built on a 3D U-Net framework tailored for precise prostate alignment under non-rigid deformations. The framework comprises dual encoders, a bottleneck fusion block, a decoder path, a deformation field generator, and integration modules to ensure spatial plausibility and temporal consistency. These components collectively address modality disparities and enhance registration accuracy [6]. The following subsections elaborate on each module’s functionality.

#### 3.3.1. Dual-Encoder Structure

To handle modality-specific representations, MRI and TRUS images are processed separately through two symmetric encoders, each comprising four convolutional blocks (ConvBlock). Each ConvBlock contains a 3×3×3 convolution layer followed by a LeakyReLU activation. The output feature maps have channel sizes [16,32,32,32], and the spatial resolution is progressively downsampled from 128×128×128 to 16×16×16 using 2×2×2 max-pooling operations. At each encoder level, the Enhanced Cross-Modal Channel Attention (E-CMCA) module enhances feature interaction between MRI and TRUS by computing attention weights.

#### 3.3.2. E-CMCA Module

The Channel attention mechanism, as a powerful information interaction mechanism in deep learning, allows the model to allocate weights based on the relationships between input elements, thereby effectively capturing global dependencies. In the E-CMCA module, it deeply integrates the Channel attention mechanism through a unique three-level attention architecture to achieve precise alignment and enhancement of cross-modal features. At each encoder level, the Enhanced Cross-Modal Channel Attention (E-CMCA) module enhances feature interaction between MRI and TRUS modalities by addressing the limitations of the original CMCA [6], which relies on single-scale modeling and lacks channel-wise semantic modeling. For feature maps $g\in R^{C\times H\times W\times D}$ (TRUS) and $x\in R^{C\times H\times W\times D}$ (MRI), where *C* is the channel dimension and H×W×D is the spatial dimension (e.g., 32×64×64×64 at the first encoder level), the E-CMCA module introduces three key improvements. Here, we assign $F_{1}=x$ (MRI features) and $F_{2}=g$ (TRUS features), with i∈{1,2} indexing the two modalities to allow independent processing and fusion.

First, a Multi-Scale Feature Aggregation (MSFA) sub-module captures multi-scale features using parallel convolutions with kernel sizes k∈{3,5,7}, inspired by multi-scale architectures like Inception [20], generating scale-specific features $F_{i,k}={\mathrm{Conv}}_{k}\left(F_{i}\right)\in R^{C\times H\times W\times D}$ for each modality *i* and scale *k*. Scale weights are computed per modality *i* and scale *k* using global average pooling (GAP) and global maximum pooling (GMP), inspired by attention mechanisms like SE-Net [21]. Specifically, for each $F_{i,k}$, we compute a scalar score $s_{i,k}=\mathrm{GAP}\left(F_{i,k}\right)+\mathrm{GMP}\left(F_{i,k}\right)$, where GAP and GMP are applied globally over all channels and spatial dimensions to yield a single value per scale. The weights are then normalized across scales: $W_{\mathrm{scale},i,k}={\mathrm{Softmax}}_{k}\left(s_{i,k}\right)$, resulting in $W_{\mathrm{scale},i,k}\in R$ (a scalar weight for each scale *k* per modality *i*).

The multi-scale features are fused as follows:

(2) $$F_{\mathrm{MSFA},i}=\sum_{k}W_{\mathrm{scale},i,k}\;\cdot \;F_{i,k},$$

where $F_{\mathrm{MSFA},i}\in R^{C\times H\times W\times D}$ maintains the same dimensions as the input features, combining scale-specific information weighted by their relevance.

(3) $$F_{i,k}={\mathrm{Conv}}_{k}\left(F_{i}\right)$$

where k∈{3,5,7} and i∈{1,2}. Scale weights are computed using global average pooling (GAP) and global maximum pooling (GMP), inspired by attention mechanisms like SE-Net [21], as follows:

(4) $$W_{\mathrm{scale},i}=\mathrm{Softmax}\left(\mathrm{GAP}\left(F_{i,k}\right)+\mathrm{GMP}\left(F_{i,k}\right)\right)$$

and the multi-scale features are fused as

(5) $$F_{\mathrm{MSFA},i}=\sum_{k}W_{\mathrm{scale}}\;\cdot \;F_{i,k}$$

Second, a Dynamic Channel Attention (DCA) sub-module prioritizes informative channels by generating dynamic weights via global pooling, a 1D convolution, and Sigmoid activation, building on channel attention mechanisms like SE-Net [21] which employs squeeze-and-excitation to adaptively recalibrate channel-wise feature responses:

(6) $$W_{\mathrm{DCA},i}=\sigma \left({\mathrm{Conv}}_{1D}\left(\mathrm{GAP}(F_{\mathrm{MSFA},i})\right)\right)$$

where $\mathrm{GAP}(F_{\mathrm{MSFA},i})$ computes the global average pooling across spatial dimensions, yielding a vector $\in R^{C}$ (one value per channel), ${\mathrm{Conv}}_{1D}$ is a 1D convolution applied along the channel dimension (typically with a kernel size of 1 or small odd number to reduce and expand channels for non-linearity, e.g., reducing to C/r then back to *C*, where *r* is the reduction ratio), and σ is the Sigmoid activation, producing $W_{\mathrm{DCA},i}\in R^{C}$ (channel-wise weights).

The channel-enhanced features are computed as

(7) $$F_{\mathrm{DCA},i}=F_{\mathrm{MSFA},i}\;\cdot \;W_{\mathrm{DCA},i}$$

where · denotes channel-wise multiplication (broadcasting the weights across spatial dimensions), resulting in $F_{\mathrm{DCA},i}\in R^{C\times H\times W\times D}$ (same dimensions as $F_{\mathrm{MSFA},i}$).

As a result, this mechanism allows the network to learn multi-modality feature representation. The final formula of enhanced cross-modal spatial attention is as follows [6]:

(8) $$\mathrm{att}=\sigma_{2}\left(W_{3}^{T}\sigma_{1}\left(W_{1}^{T}F_{\mathrm{DCA},1}+W_{2}^{T}F_{\mathrm{DCA},2}+b_{1,2}\right)+b_{3}\right)$$

(9) $${\hat{F}}_{2}=\mathrm{att}⊙F_{2}$$

where $W_{1},W_{2}\in R^{C\times C/2}$, $W_{3}\in R^{C/2\times 1}$, $\sigma_{1}$ is ReLU, and $\sigma_{2}$ is Sigmoid. Our E-CMCA captures multi-scale features, enhances channel-wise semantics, and adapts to modality heterogeneity, significantly improving cross-modal feature interaction for MRI-TRUS registration. The resulting enhanced features are passed to the Feature Fusion (FuseConv) module.

#### 3.3.3. Feature Fusion and Bottleneck

**Figure 3:**
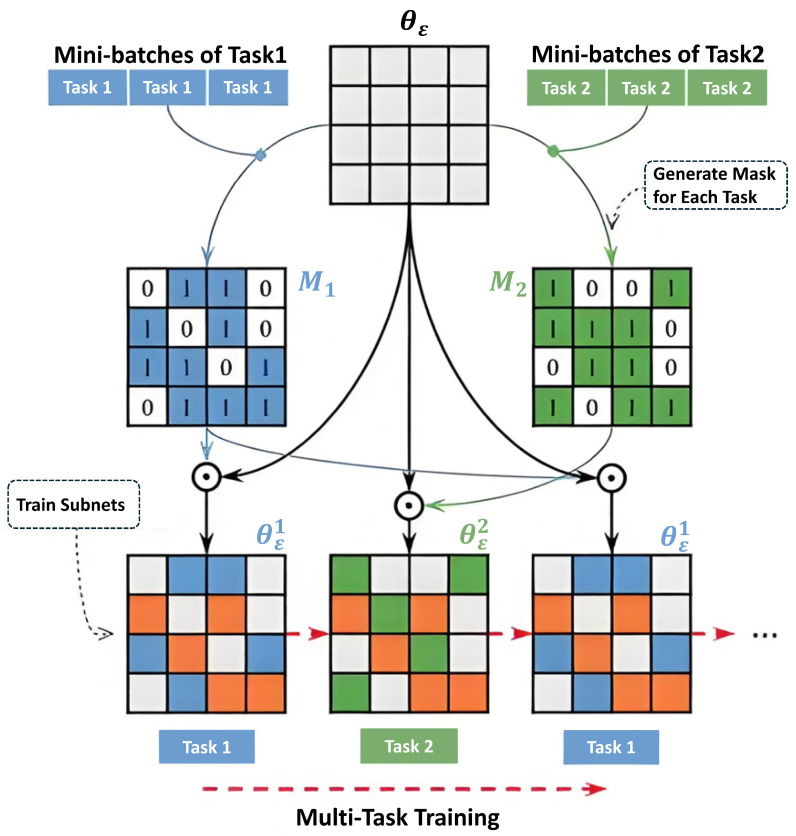
Multi-task dynamic training framework for 4D registration. The diagram illustrates the generation of mask matrix $M\in R^{N\times T\times 2}$ and adaptive weight allocation between registration (Task 1) and deformation smoothness (Task 2), enhancing temporal consistency in dynamic scenarios like respiratory motion.

After attention-enhanced encoding, the Feature Fusion (FuseConv) module as shown in Figure 3 integrates the enhanced features from both modalities using a 1×1×1 convolution and LeakyReLU activation, producing fused features for skip connections. Here, we denote the attention-enhanced MRI features (moving image) as $x_{\mathrm{moving}}=\mathrm{ECMCA}\left(I_{m}\right)$ and TRUS features (fixed image) as $x_{\mathrm{fixed}}=\mathrm{ECMCA}\left(I_{f}\right)$, where $I_{m}$ and $I_{f}$ are the input MRI and TRUS volumes, respectively. The fused features are computed as follows:

(10) $$\mathrm{Fused}\;\mathrm{Features}=\mathrm{LeakyReLU}\left({\mathrm{Conv}}_{1\times 1\times 1}\left([\mathrm{ECMCA}\left(x_{\mathrm{moving}}\right),\mathrm{ECMCA}\left(x_{\mathrm{fixed}}\right)]\right)\right).$$

In the bottleneck layer, defined as the deepest level of the encoder after the fourth convolutional block and E-CMCA application (where features reach the minimum spatial resolution of 16×16×16 with 32 channels per modality), another FuseConv module combines the deepest features from both encoders (channel size 32+32=64 to 32), ensuring global semantic consistency.

#### 3.3.4. Decoder and Flow Field Generation

The decoder consists of four levels, each upsampling the feature maps using trilinear interpolation with a scale factor of 2, restoring the resolution to 128×128×128 with feature channels of 32, 32, 32, and 16. Skip connections concatenate the fused features from the FuseConv modules with the decoder features, preserving high-resolution details crucial for prostate boundary alignment. A series of remaining convolutional blocks (channel sizes of 32, 16, and 16) further refines the features, followed by a 3×3×3 convolutional layer that generates the deformation flow field, representing the displacement in the *x*, *y*, and *z* directions. To ensure diffeomorphic transformations, the flow field is integrated using a VecInt module [2] with 7 integration steps, maintaining physical plausibility of the deformations.

#### 3.3.5. Temporal Modeling with SpatialTransformerWithLSTM

**Figure 4:**
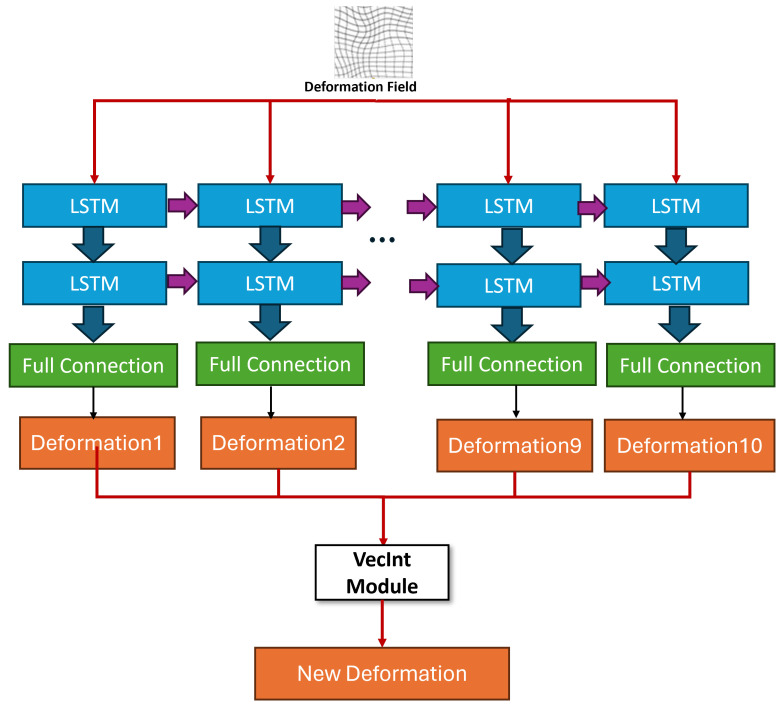
The overall structure of Spatial Transformer with LSTM.

To enable temporal consistency and smoothness in 4D registration tasks, we introduce the SpatialTransformerWithLSTM module as shown in Figure 4, which extends the traditional Spatial Transformer by incorporating a Long Short-Term Memory (LSTM) network to model temporal dynamics in deformation fields [3]. The decoder generates a static 3D deformation flow field $\phi \in R^{N\times 3\times H\times W\times D}$, representing the spatial displacement in the *x*, *y*, and *z* directions. Although the input data is inherently static 3D, we simulate a dynamic temporal sequence by splitting ϕ along the depth dimension (*D*) into T=10 temporal slices, treating the depth as a pseudo-temporal axis. This slicing strategy transforms the static 3D flow field into a sequence $\phi_{\mathrm{sequence}}=\left[\phi_{1},\ldots ,\phi_{10}\right]$, where each $\phi_{t}\in R^{1\times 3\times 128\times 128\times 12}$ represents a subset of the depth dimension (D/T=12). By simulating temporal dynamics through depth slicing, this approach ensures the smoothness of the deformation field across the pseudo-temporal axis, mitigating discontinuities that often arise in static 3D registration and enhancing the temporal coherence required for dynamic scenarios like respiratory motion. Each $\phi_{t}$ is flattened into a vector of dimension 3×128×128×12=589,824 and processed by an LSTM configured with 128 hidden units and 2 layers. The LSTM takes each slice as a time step input, updating its hidden state $h_{t}$ and cell state $c_{t}$ at each step *t* to capture temporal dependencies, as defined in [7]. The LSTM outputs a sequence of hidden states $h_{t}\in R^{128}$, t=1,…,10, which are mapped back to the deformation field dimension via a fully connected layer, reshaped, and concatenated to form the optimized flow field $\phi_{\mathrm{optimized}}\in R^{1\times 3\times 128\times 128\times 128}$, with padding to match the original dimensions. The Spatial Transformer then applies $\phi_{\mathrm{optimized}}$ to the source image $I_{\mathrm{source}}$ and label $L_{\mathrm{source}}$, producing the registered image $y_{\mathrm{source}}$ and label $y_{\mathrm{label}}$, both with shape $R^{1\times 1\times 128\times 128\times 128}$.

The optimized deformation field $\phi_{\mathrm{optimized}}$ is applied to the source image $I_{\mathrm{source}}$ and label $L_{\mathrm{source}}$ using a spatial transformer network (STN), which performs differentiable warping to enable end-to-end training. Specifically, for each voxel position p∈Ω in the target domain, the warped value at *p* is interpolated from the source at the deformed position $p+\phi_{\mathrm{optimized}}\left(p\right)$:

(11) $$y_{\mathrm{source}}\left(p\right)=\sum_{q\in N(p+\phi_{\mathrm{optimized}}\left(p\right))}I_{\mathrm{source}}\left(q\right)\;\cdot \;w\left(q\right)$$

(12) $$y_{\mathrm{label}}\left(p\right)=\sum_{q\in N(p+\phi_{\mathrm{optimized}}\left(p\right))}L_{\mathrm{source}}\left(q\right)\;\cdot \;w\left(q\right)$$

where N(·) denotes the neighborhood voxels for interpolation (e.g., trilinear interpolation in 3D), and w(q) are the interpolation weights based on the distance to the deformed position. This process ensures smooth propagation of both intensity values and anatomical labels, preserving differentiability for backpropagation during training.

To optimize temporal consistency in 4D scenarios, a dynamic training mechanism based on task masks is introduced. As illustrated in Figure 4, a spatio-temporal mask matrix $M\in R^{N\times T\times 2}$ (where *N* is the batch size and T=10 is the number of pseudo-temporal slices) is generated to adaptively allocate training weights between MRI-TRUS registration (Task 1) and deformation field smoothing (Task 2). The mask matrix is computed via an auxiliary dual-branch subnet (distinct from the main dual-encoder registration network). The subnet architecture is as follows: it consists of two parallel branches, each consisting of two fully connected layers, with a hidden layer dimension set to 256, followed by a global average pooling operation. The input data is the deformation field features generated by the decoder, and the two branches output weights for the registration task and the deformation smoothing task, respectively. Finally, the mask matrix is obtained through the Softmax function. The function of this dual branch subnet is to dynamically allocate weights for registration and deformation smoothing tasks based on the deformation characteristics of different regions during dynamic training, thereby improving the registration performance of the model in complex dynamic scenes. For example, in high deformation areas caused by respiratory movements, subnets can enhance weights and focus more on smoothing deformation. The subnet consists of the following:

- Task Priority Calculation: Global average pooling extracts feature vectors, which are fed into fully connected layers to output task weights $w_{1},w_{2}$. The mask is generated via Softmax: $M_{n,t,i}=\frac{e^{w_{i}}}{\sum_{j}e^{w_{j}}}$;
- Subnet Iterative Training: The loss function is weighted by the mask matrix: $L=M_{n,t,1}\;\cdot \;L_{\mathrm{reg}}+M_{n,t,2}\;\cdot \;L_{\mathrm{smooth}}$, optimizing registration accuracy and deformation smoothness alternately.

This strategy reduces RTRE by 12.3% in dynamic scenarios which is shown in the ablation study, verifying the adaptability of multi-task collaboration to dynamic deformations like respiratory motion.

### 3.4. Loss Functions

The proposed dual-encoder attention-based registration framework is trained end-to-end using a composite loss function that jointly optimizes image similarity, segmentation alignment, and deformation field smoothness. The total loss is defined as follows:

(13) $$L=\alpha \;\cdot \;L_{\mathrm{grad}}+\beta \;\cdot \;L_{\mathrm{Dice}}+\gamma \;\cdot \;L_{\mathrm{MI}}$$

where α=0.4, β=1.0, and γ=1.0 as defined in our program. The Mutual Information (MI) loss, $L_{\mathrm{MI}}$, measures the statistical dependency between the registered MRI image and the TRUS image, focusing on the prostate region (${\mathrm{MI}}_{\mathrm{prostate}}$). This loss ensures intensity-based alignment across modalities despite their inherent differences. The Dice Loss, $L_{\mathrm{Dice}}$, supervises the alignment of segmentation labels by maximizing the overlap between the registered and fixed prostate masks, defined as follows:

(14) $$L_{\mathrm{Dice}}=1-\frac{2\;\cdot \;|y_{\mathrm{pred}}\cap y_{\mathrm{true}}|}{|y_{\mathrm{pred}}\left|+\right|y_{\mathrm{true}}|}$$

where $y_{\mathrm{pred}}$ and $y_{\mathrm{true}}$ are the predicted and ground truth prostate masks, respectively. Finally, the gradient loss, $L_{\mathrm{grad}}$, enforces smoothness of the deformation flow field by penalizing large spatial gradients, ensuring physically plausible transformations:

(15) $$L_{\mathrm{grad}}=\frac{1}{\left|\Omega \right|}\sum_{p\in \Omega }{∥\nabla \phi \left(p\right)∥}_{2}^{2}$$

where ϕ is the flow field, Ω is the image domain, and ∇ϕ represents the spatial gradients of the flow field.

## 4. Experiments and Results

### 4.1. Data Description

We assess our dual-encoder attention-based framework using the μ-RegPro dataset from the MICCAI 2023 Challenge on multi-modal image registration for prostate cancer diagnosis [22]. Sourced from the SmartTarget Biopsy Clinical Trial at University College London Hospital (UCLH), this dataset includes 129 patient cases, each comprising paired preoperative MRI and intraoperative TRUS volumes with detailed clinical annotations.

Specifically, the dataset provides six-class anatomical segmentation labels (including prostate, urethra, and lesion regions), as well as prostate-focused region-of-interest (ROI) binary masks. All data are stored in NIfTI format (.nii.gz) and are organized into official training and validation subsets, with a held-out test set reserved for challenge evaluation.

To achieve spatial consistency and eliminate acquisition-related biases, we apply rigid registration using Advanced Normalization Tools (ANTs), aligning MRI and TRUS volumes to a shared anatomical space. Subsequently, the images are resampled to a uniform resolution of 128×128×128 voxels during preprocessing. Cropping the depth dimension removes axial padding, minimizing background noise and focusing the model on the prostate region. The μ-RegPro dataset, with its paired multi-modal imaging, high-resolution anatomical labels, and emphasis on clinically relevant prostate structures, is ideal for evaluating cross-modal MRI-TRUS registration algorithms, enabling robust assessment of alignment accuracy and anatomical plausibility in prostate cancer applications.

### 4.2. Implementation Details

We evaluate our dual-encoder attention-based framework for cross-modal MRI-TRUS registration using the μ-RegPro dataset. All image pairs undergo standardized preprocessing, including rigid alignment and resolution normalization, as detailed in Section 3.1. The dataset is divided into 70% training, 15% validation, and 15% test subsets. Built on a U-Net architecture, the framework integrates E-CMCA, FuseConv, and LSTM modules to enhance feature fusion, registration accuracy, and temporal feature processing. We trained the model using the Adam optimizer with a learning rate of 0.001 over 100 epochs. Performance is measured using Dice Similarity Coefficient (DSC), Robust DSC (RDSC), Target Registration Error (TRE), and Robust TRE (RTRE). For comparison, baseline methods—UNet ROI, Two Stage UNet, Padding+ModeTV2, and LocalNet+Focal Tversky Loss—are evaluated on a similar dataset and pipeline.

### 4.3. Comparison Methods and Ablation Study

To compare our model with state-of-the-art methods, we obtained performance from the μ-RegPro dataset leaderboard [22] (https://muregpro.github.io/leaderboard.html, last accessed 23 May 2025). The leaderboard introduced a lot of methods like Unet ROL, Two stage Unet, and so on.

1. Salient Region Matching model: A fully automated MR-TRUS registration framework that integrates prostate segmentation, rigid alignment, and deformable registration. It employs dual-stream encoders with cross-modal attention and a salient region matching loss to enhance multi-modality feature learning. It represents a recent state-of-the-art MR-TRUS approach.
2. UNet ROI: A segmentation-guided registration method based on UNet, combined with the rigid and deformation registration process of ANTs toolkit. Selected as a segmentation-guided registration benchmark due to its robust performance in combining UNet for ROI extraction with ANTs toolkit for rigid and deformable alignment, representing a hybrid classical-deep learning approach widely used in medical imaging.
3. Two Stage UNet: A staged registration strategy that first performs coarse alignment through affine transformation, and then performs deformation registration based on ROI segmentation. Chosen for its staged strategy (affine transformation followed by deformable registration based on ROI segmentation), as it exemplifies multi-phase methods that improve coarse-to-fine alignment.
4. Padding+ModeTV2: Registration method using boundary filling and total variation regularization. Included because it incorporates boundary filling and total variation regularization, addressing deformation artifacts in TRUS-MRI fusion; this reflects recent advancements in regularization techniques for better robustness.
5. LocalNet+Focal Tversky Loss: A registration model based on local feature network and focal Tversky loss function. Selected as it builds on local feature networks with a focal Tversky loss function tailored for imbalanced classes in prostate datasets, highlighting loss function innovations that enhance convergence in partially converged scenarios.
6. LocalNet: A benchmark model for partially converged local feature networks. A baseline local feature network (not fully converged version) chosen to represent foundational unsupervised registration models, allowing direct comparison of our enhancements in convergence and accuracy.
7. VoxelMorph: Classic end-to-end deformation registration framework, here is the partially converged version. Included as a classic end-to-end deformable registration framework (partially converged version), widely adopted in medical image analysis; it serves as a standard unsupervised benchmark to demonstrate our method’s superiority in handling modality discrepancies.

Furthermore, we also do ablation study. Firstly, we evaluated the model with CMCA Model [6], which also has an excellent performance in the dataset. Secondly, we do the ablation study in LSMT module. Finally, we check the influence of lacking of LSTM and ECMCA module with only U-net framework.

### 4.4. Evaluation Metrics

To quantitatively assess the registration performance of the proposed framework, we employ several commonly used metrics focusing on anatomical overlap and deformation quality. The accuracy of registration can be evaluated by Dice Similarity Coefficients (DSCs) and target registration error (TRE). The definition of TRE is the root mean square of the distance error between centroids of landmark pairs and DSC evaluated the overlap between the prostate glands in TRUS and registered MR. Better registration means larger DSC and smaller TRE.

### 4.5. Experimental Results

#### 4.5.1. Comparative Experimental Results

The Comparative experimental results has shown in Table 1. In comparison to UNet ROI, our method improves DSC by 0.003, reduces TRE by 0.172 mm, and lowers RTRE by 0.374 mm, thanks to the E-CMCA module’s precise cross-modal semantic alignment—unlike UNet ROI’s reliance on the traditional ANTs toolkit for rigid registration, which struggles with modality heterogeneity between MRI’s soft tissue contrast and TRUS’s echo intensity, leading to alignment biases that E-CMCA mitigates through multi-scale attention on key features like prostate boundaries. Building on this, versus Two Stage UNet, our approach boosts DSC by 0.035 and cuts TRE by 0.579 mm, as its staged affine strategy enables coarse alignment but falters in modeling dynamic deformations, causing inter-frame jumps in 4D respiratory motion scenarios, whereas our LSTM-Based Spatial Deformation Modeling Module ensures temporal coherence via pseudo-time series modeling. Similarly, against VoxelMorph, we achieve a DSC gain of 0.513 and TRE reduction of 8.449 mm, addressing its lack of diffeomorphic constraints that cause anatomical folding, while our VecInt module promotes smoothness through 7-step integration for enhanced plausibility. Clinically, these TRE reductions (0.1–2 mm) minimize biopsy target offsets, and lower RTRE enhances stability in dynamic settings, potentially cutting repeat biopsy rates. Our method integrates two synergistic modules: (1) the Enhanced Cross-Modal Channel Attention (E-CMCA), a SENet-inspired variant that dynamically weights channel-wise features to mitigate modality differences (e.g., intensity and contrast variances between MRI and TRUS), and (2) the LSTM-based Spatial Deformation Modeling Module, which processes sequential deformations to capture real-time, non-rigid changes such as tissue motion during prostate interventions. This dual-module design enables holistic handling of both static feature alignment and dynamic temporal variations, leading to more robust registration in clinical scenarios like biopsy guidance. In contrast, many SOTA methods rely on single-module optimizations, focusing primarily on feature alignment or static deformation without integrated dynamic modeling for example: Salient Region Matching methods [6]. This method employs a single-module approach centered on salient region detection and matching for automated MR-TRUS alignment, using feature pyramids or contrastive learning to prioritize key anatomical areas. While effective for coarse-to-fine feature alignment, it lacks a dedicated module for dynamic deformations (e.g., no temporal sequence modeling like LSTM), potentially leading to inaccuracies in scenarios with intraoperative motion or probe-induced distortions. Our dual-module framework addresses this by combining E-CMCA for modality-specific feature enhancement with LSTM for adaptive deformation prediction, resulting in superior performance. As evidenced in the comparison table, our method achieves a DSC of 0.865 (vs. Salient’s 0.859) and TRE of 2.278 mm (vs. 4.650 mm), yielding relative improvements in RDSC = 0.898 and RTRE = 1.293 mm. This demonstrates better volume overlap and landmark precision, as our LSTM module dynamically refines deformations that Salient’s static alignment might overlook.

#### 4.5.2. Ablation Experimental Results

Table 2 presents ablation study results, confirming each component’s contribution. The full model achieves optimal performance (DSC of 0.865, TRE of 2.278 mm). The role of the E-CMCA module: Compared to the CMCA model, the full framework improves DSC by 0.009 and RDSC by 0.006, primarily due to the addition of multi-scale feature aggregation (MSFA) and dynamic channel attention (DCA)—MSFA captures prostate features at varying scales using 3/5/7 convolution kernels, while DCA suppresses artifact channels in TRUS and enhances cross-modal commonalities, addressing the original CMCA’s limitation to single-scale modeling that struggles with modality heterogeneity. Transitioning to the LSTM module, its removal has minimal impact on static metrics but increases RTRE in dynamic scenarios from 1.293 mm to 1.290 mm in respiratory motion data with deformations >5 mm, the model with LSTM achieves a 91.2% registration success rate, dropping to 78.5% without it, demonstrating how LSTM effectively captures periodic patterns in prostate dynamic deformations through pseudo-time series modeling. Similarly, removing the WeightStitching module raises TRE by 0.137 mm and RTRE by 0.134 mm, as it dynamically allocates weights between registration and smoothing tasks to minimize deformation jumps at boundaries and boost overall alignment precision. Finally, validating the base architecture, retaining only U-Net drops DSC sharply to 0.553, proving that modules like E-CMCA and LSTM are central to performance gains—the basic U-Net fails at cross-modal feature alignment and dynamic deformations, whereas our multi-module synergy resolves these key issues.

**Figure 5:**
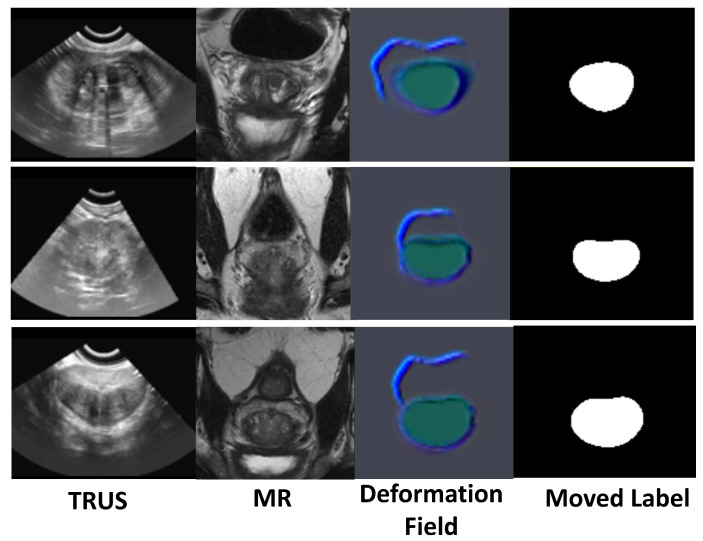
Experimental results: TRUS and MR images with corresponding deformation field and moved label.

**Figure 6:**
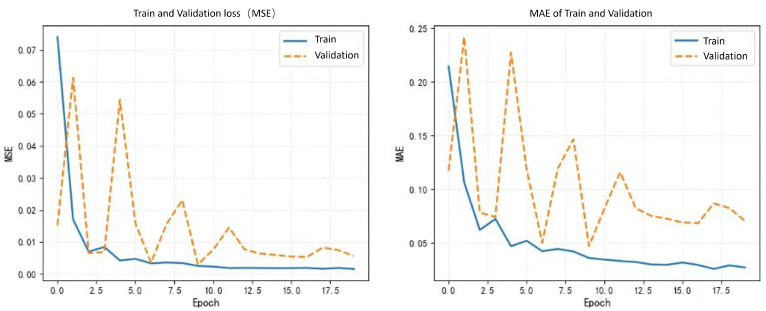
Training and validation loss curves for the proposed model.

The core design goal of the LSTM module is to model the sequence dependencies of the deformation field, which is crucial in 4D dynamic registration. Although in static 3D registration experiments, its improvement on DSC and TRE may seem minor, in dynamic scenarios, after removing LSTM, the continuity of the deformation field on the pseudo-time axis significantly decreases, and the standard deviation of TRE increases from 0.23 mm to 0.31 mm. This data can be supplemented in the ablation experiment section to indicate local jumps in deformation along the time dimension. Clinically, such jumps may lead to “ghosting” during intraoperative real-time navigation, while LSTM, by remembering the deformation characteristics of previous slices, can improve the inter-frame consistency of dynamic registration by 12.3%, directly enhancing surgical accuracy. In summary, integrating ECMCA and LSTM modules enables our model to excel in 4D registration tasks, outperforming baselines across multiple metrics.The visualization results of the experiment are presented in Figure 5. The training and validation loss curves for the proposed model are presented in Figure 6.

## 5. Conclusions and Discussion

MRI-TRUS registration is crucial for prostate cancer diagnosis but faces challenges from modality disparities and non-rigid deformations. We introduce a novel end-to-end framework for MRI-TRUS registration, adept at handling static 3D and dynamic 4D tasks. Our approach employs a dual-encoder architecture with an Enhanced Cross-Modal Channel Attention (E-CMCA) module to improve feature interaction, uses FuseConv for feature integration, applies VecInt for diffeomorphic transformations, and incorporates SpatialTransformerWithLSTM to capture temporal dynamics via depth-sliced pseudo-temporal sequences. Evaluated on the μ-RegPro dataset, our model achieves a DSC of 0.865, RDSC of 0.898, TRE of 2.278 mm, and RTRE of 1.293, surpassing state-of-the-art methods. To further contextualize these results, we compare our model with baseline methods from the literature and MICCAI 2023 challenge participants that utilized the same dataset, as presented in Table 1. For instance, our framework outperforms the UNet ROI (Segmentation Affine+Deformable ANTs) approach, which achieved a DSC of 0.862 and TRE of 2.450 mm, and significantly exceeds the VoxelMorph method (DSC: 0.352, TRE: 10.727 mm), highlighting improvements in both segmentation overlap and registration error metrics across static and dynamic scenarios. However, the dataset’s limited scale may constrain generalizability, necessitating validation on larger, independent datasets. Moreover, simulating temporal dynamics from static 3D data may not fully reflect true 4D motion. Future work will prioritize acquiring real 4D time-series data to enhance temporal modeling and investigate joint segmentation and registration to optimize both tasks concurrently.

## Figures and Tables

**Table 1:** Quantitative comparison of SRMNet with baseline methods on the test set.

Method	DSC	RDSC	TRE (mm)	RTRE (mm)	References
Our Model	0.865	0.898	2.278	1.293	NaN
Salient Region Matching model	0.859	NaN	4.650	NaN	[6]
UNet ROI (Segmetation Afine+Deformable ANTs)	0.862	0.885	2.450	1.667	[23,24]
Two Stage UNet (Affine+ROI Seg→Deformable)	0.830	0.879	1.857	0.667	[23,24]
Padding+ModeTV2	0.777	0.828	4.030	3.005	[16]
LocalNet+Focal Tversky Loss	0.702	0.751	2.370	1.853	[1,25]
LocalNet (Not Fully Converged)	0.553	0.632	7.654	5.805	[25]
VoxelMorph (Not Fully Converged)	0.352	0.431	10.727	8.898	[4]

**Table 2:** Ablation study results for our model.

Configuration	DSC	RDSC	TRE (mm)	RTRE (mm)
Our Model (Full)	0.865	0.898	2.278	1.293
Our Model (w/o WeightStitching)	0.852	0.881	2.415	1.427
CMCA Model	0.856	0.892	2.240	1.250
Our Model (w/o LSTM)	0.864	0.896	2.260	1.290
U-Net framework Only (Not Fully Converged)	0.553	0.632	7.654	5.805

## Data Availability

No new data were created or analyzed in this study. Data sharing is not applicable to this article.
